# Healthy ageing in Isan-Thai culture—A phenomenographic study based on older persons’ lived experiences

**DOI:** 10.3402/qhw.v11.29463

**Published:** 2016-03-08

**Authors:** Pornpun Manasatchakun, Pleumjit Chotiga, Åsa Roxberg, Margareta Asp

**Affiliations:** 1 School of Health, Care and Social Welfare, Mälardalen University, Eskilstuna-Västerås, Sweden; 2 Boromarajonani College of Nursing Chiang Mai, Chiang Mai, Thailand

**Keywords:** Caring science, healthy ageing, Isan-Thai culture, lifeworld theory, phenomenography

## Abstract

Healthy ageing is a concept that concerns older persons’ quality of life and is a key factor in promoting well-being. The older population in Thailand is growing. Isan (a region of north-eastern Thailand) has been reported as having one of the most rapidly increasing older populations in the country. In order to care for and promote the health of older people, healthcare providers should understand how healthy ageing is perceived by this target group. Although healthy ageing has been studied in different contexts as well as perspectives, no studies have previously focused on older persons’ experiences of healthy ageing from a lifeworld perspective in Isan-Thai. Therefore, the aim of this study is to describe older persons’ qualitatively different conceptions of healthy ageing in Isan-Thai culture. A phenomenographic approach with an epistemological base in lifeworld theory was used to disclose the various ways to conceptualize healthy ageing. Individual, qualitative interviews were conducted with 17 people aged 60 and above who live in Isan-Thai. The findings of this study revealed three categories of descriptions: “being independent in dependence,” “being at peace,” and “being a valuable person.” This study also found family members, friends, healthcare providers, and religion important to healthy ageing in the Isan-Thai culture. Understanding how older people conceptualize healthy ageing is valuable for healthcare providers. They can apply these findings regarding healthy ageing in their fieldwork when caring for older people.

Thailand is facing an increasing older population which is ranked the second fastest growing in Southeast Asia (Sasat & Bowers, 2013; United Nations, 2011). The average length of life has risen for both men and women when considering life expectancy in the region (United Nations, 2015). The rate of morbidity is increasing while the mortality rate is falling (Knodel & Chayovan, 2008). Older persons are likely to face greater risks, which eventually lead to chronic diseases and even disabilities (Knodel & Chayovan, 2008). Additionally, an increase in the morbidity rate can increase healthcare costs (Abegunde, Mathers, Adam, Ortegon, & Strong, 2007). The conclusion of this development is that for both human and economic reasons it is important to maintain health in old age.

The north-eastern region of Thailand, “Isan,” is one of the regions where the rapid increase in the number of older people can be most clearly seen (UNFPA, 2006). Isan is populated by people of Lao descent as it shares a border with Laos and Cambodia. The World Bank (2005) reported that this region is the poorest region in Thailand. Isan exhibits the effects of modernization on older people (Caffrey, 1992). Isan lifestyle, as in other Thai regions, is influenced by Buddhism, the national religion of Thailand. A predominant fundamental Buddhist principle is that children take care of their parents. This is a reciprocal relationship because parents provide care for their children in their childhood (Caffrey, 1992; Sasat & Bowers, 2013). However, Isan exhibits the effects of modernization on older people (Caffrey, 1992) and social changes have altered the Isan custom. Sudnongbua, LaGrow, and Boddy (2010) found that children in Isan tend to migrate to the cities to find an economically advantaged position and their old parents can feel abandoned by them.

Healthy ageing has become a key factor in preserving health and promoting well-being for older persons. This concept has been adopted in various research areas and conducted in a variety of perspectives (Clarke & Nieuwenhuijsen, 2009; Franco et al., 2009; Hansen-Kyle, 2005; Seib, Anderson, & Lee, 2014; Thiamwong, McManus, & Suwanno, 2013). Accordingly, several studies showed that healthy ageing is influenced by physiological, psychological, social, and, especially, cultural factors (Danyuthasilpe, Amnatsatsue, Tanasugarn, Kerdmongkol, & Steckler, 2009; Thiamwong et al., 2008; Touhy & Jett, 2010). Hansen-Kyle (2005) found that a medical perspective emphasizes healthy ageing as an ability to maintain physical, psychological, and social function, and it prevents older persons from getting chronic diseases. Regarding sociological and psychological perspectives, healthy ageing focuses on one's attitude, autonomy, support structure, and individual independence as fundamental aspects of healthy ageing (Hansen-Kyle, 2005). These perspectives emphasize the process of changing in ageing, rather than describing what healthy ageing means from the older people's own experiences. However, from a nursing perspective, healthy ageing has been focused on the individual's ability to perform normal daily activities, social and psychological aspects (Hansen-Kyle, 2005). In Thailand, according to nursing research, healthy ageing focuses on the interaction among physical, mental, environmental, and religious dimensions (Danyuthasilpe et al., 2009; Thanakwang & Soonthorndhada, 2011; Thiamwong et al., 2008). These studies about healthy ageing concern older persons’ perspectives to develop a model of healthy ageing. Thiamwong et al. (2013) described the model of healthy ageing which comprises three themes: normality which means living a normal life, nature which comprises living a simple and natural life, and dharma which focused on practices in Buddhism. Another study of Thanakwang, Soonthorndhada, and Mongkolprasoet (2012) developed a healthy ageing model which comprises independence, no chronic diseases, a positive aspect of psycho-emotional outlook, and social contribution.

There is, however, no research found concerning older persons’ experiences of healthy ageing in the Isan-Thai culture although there are some studies of healthy ageing in Thailand. In order to establish a care that contributes to healthy ageing, which is the goal of caring (Wikberg & Eriksson, 2008), healthcare providers should take into account older people's view points and include what healthy ageing means from older person's perspectives.

## Aim

The aim of this study was to describe older persons’ qualitatively different conceptions of healthy ageing in Isan-Thai culture.

## Method

This interview study has a phenomenographic approach, based in the lifeworld theory as recommended by Ashworth and Lucas (1998). The motive was to gain a deeper insight into the variations of the conception of healthy ageing from older people's lifeworlds. The lifeworld perspective is the world as it is experienced and perceived by humans (Dahlberg, Dahlberg, & Nyström, 2008). The aim of lifeworld theory and phenomenography is to describe how human beings give meaning to their world. Human beings and their existence are accordingly apprehended as a whole (Dahlberg et al., 2008; Merleau-Ponty, 2005/1995). Therefore, man is an inalienable unit (Merleau-Ponty, 2005/1995). A phenomenographic approach is to discover the qualitative differences in the conceptions of the phenomenon as expressed in the data from a second-order perspective (Marton, 1981; Marton & Booth, 1997). Marton (1981) described a second-order perspective as focusing on how people experience phenomena in the world.

### Participants and study setting

According to the World Health Organization (WHO, 2015), older adults mean people who are 65 years and older, which is accepted by most developed countries. However, the United Nations uses other criteria to classify older adults and defines them as people who are 60 years and older (WHO, 2015). These definitions show that the age of older persons is around the age of retirement (Knodel & Chavan, 2008; WHO, 2015). According to the Thai government, Thai older persons are defined as people who are aged 60 or older (Gray, Pattaravanich, Chamchan, & Prasartkul, 2015; Knodel, Prachuabmoh, & Chayovan, 2013). This definition refers to the retirement age in Thailand. Because the researchers needed to include the perspectives of older persons in Isan-Thai, the participants in this study were people aged 60 or older. The setting was in Udon Thani province in the Isan region of Thailand. This province was selected purposively because it is one of the provinces where there is a rapidly growing older population. Furthermore, this province showed characteristics of both rural and urban areas. In this study, according to the administrative classification in the Thai government (Ircha, 2002) and the United Nations (ESCAP, 2013), urban areas refer to municipal areas and rural areas mean non-municipal areas. The purposive sampling approach was used to help the researcher obtain variation in the data. The selection of older persons was based on recommendations by public health professionals or nurses in the community and a leader of a club for older people. In order to maximize variation, participants were chosen strategically based on age, marital status, education level, health status, and geographical location. Inclusion criteria for the selection of participants were 60 years and above, able to speak Thai, and willing to participate. Exclusion criteria were older people who suffered from a psychiatric diagnosis that might interfere with the participants’ ability to communicate. In total, 17 older people (14 females and 3 males) were chosen who agreed to participate in the research project. All of them were Buddhist. The mean age of the 17 participants was 72.8 years with age ranging from 66 to 85 years. Five of participants were diagnosed with chronic diseases such as diabetes, gout, and hypertension. Ten participants had no formal education. Six had an elementary education and one had university-level education. Eleven were married and living with their partner and some of them were also living with children and grandchildren. Five were widowed and lived with their children or grandchildren. One of them was widowed and lived alone. Ten of the participants lived in urban areas and seven lived in rural areas.

### Data collection

Data were collected during August and September 2013. A list of older people living in each district was obtained from healthcare professionals and a leader of a club for older people. Then the researcher contacted older people who met the inclusion criteria in both rural and urban areas. A pilot interview was conducted to test the interview questions (Green, 2005). The interview was specifically designed to be a conversation where knowledge is elicited through the interaction between the researcher and the participants (Liamputtong, 2009). A semi-structured interview with open-ended questions was conducted in order to obtain information about participants’ experiences of healthy ageing and to explore their own thoughts (Dahlberg et al., 2008; Holloway & Wheeler, 2010; Marton & Booth, 1997). The opening questions of the interviews were followed by questions about their conceptions of healthy ageing, such as “What do you think about healthy ageing?”, “What makes aged people healthy, in your opinion?”, and “Do you think you are a healthy ager?” Participants who answered yes or no were asked questions in order to clarify and deepen their answers. Examples of questions included “Why do you think in that way?”, “Please give me the reasons?”, “Could you please explain it to me?”, “Could you please give me an example?”, and “What do you mean by …?” The dates and times of the interviews depended on what was convenient for the participants. The interviews were tape-recorded. The first author was able to observe and record non-verbal behaviour in the field notes, which were written during the interviews. He also took note of the participants’ body language, tone of voice, and the important points in these field notes. These observations helped the first author guide the next question in order to create a fluent conversation about the topic in hand. However, the authors did not apply these field notes in the analysis process. The length of the interview depended on each participant and ranged from 45 to 60 min, and the interviews were conducted in Thai. After interviews, the data were transcribed verbatim in Thai. Next, all transcripts were translated from Thai to English by a native licensed professional Thai-English translator in order to confirm the accuracy of the transcripts (Regmi, Naidoo, & Pilkington, 2010). After translation, transcripts were confirmed by the first and the second author from Thailand.

### Data analysis

The authors analysed the transcribed interviews as suggested by Dahlgren and Fallsberg (1991). The first step of the data analysis was familiarization. The first author read the transcripts several times to obtain an overview of the participants’ experiences and conceptions and to become familiar with the content. This step was necessary in order to check the correctness of the transcriptions. The second step, condensation, was conducted to reduce the individual answers and to find the main central parts of the dialogue by the first author. The third step, comparison, was used to select significant dialogue excerpts and compare the similarities and differences in the dialogue. All authors participated in the analysis from the third step and forward. In the fourth step similar statements were grouped together as preliminary descriptive categories. The fifth step, articulating, was used to make a description of the essence of the similarity within each group of statements. The sixth step was the labelling of the categories which were shown by constructing an appropriate linguistic expression. The last step, contrasting, was used to compare the differences and similarities in a contextual sense. Each category was made clear by means of quotations from the participants so that the categories contained a description of their unique character.

### Ethical considerations

Ethical considerations and approval were carried out by the Udon Thani Provincial Public Health Office Committee Board in Thailand and the regional ethics committee, Uppsala, Sweden (Dnr 2013/019), and followed the principles outlined in the Declaration of Helsinki (WMA, 2013). Both an information letter and a consent form were given to each participant by the first author. The purpose of the study, the process, and the time required for participating were explained to the participants. The interview time and the place were designed by the participants. It was emphasized that participation was voluntary and all participants were told that they could withdraw from the study at any time without negative consequences. The participants were informed of the possible benefits of the study. The participants who accepted to participate in the study signed an informed consent form. The identity of each participant was concealed by using a code name and their identity could not be visible in the data. Confidentiality was guaranteed in order to ensure that information about of the participants could not be recognized in the results of the study.

## Findings

The analysis resulted in three categories of description: (1) being independent in dependence, (2) being at peace, and (3) being a valuable person. These three descriptive categories are described below and elucidated by quotations from the interviews.

### Being independent in dependence

This category illustrated how healthy ageing is understood in terms of being independent in dependence. In this case, it is a belief in oneself to be able to do things without depending on others. At the same time it is also about depending on another person close to them. Some of the described situations of being independent in dependence are being able to do, or select, something by oneself although requiring some level of support from family members or friends. This is regardless of whether they currently have a disease or illness at that time which requires care from healthcare providers. The first category brings together two sub-categories which were “being able although requiring support” and “needing care from healthcare providers regardless of disease or illness.”

### Being able although requiring support

Healthy ageing means having the ability to do things for and taking care of oneself. It is about believing in oneself to do what one wants to do. It is to be free to select things which are based on the individual's beliefs. It is also a belief in one's own ability and one's knowledge to follow one's wishes in order to maintain a strong body and cognitive function. However, at the same time, it is about being satisfied with life, having a warm feeling and feeling safe in life when receiving support from others (family, friends, etc.). In other words, it is about being confident in one's own ability to live and do things independently while having others who will be there to assist if needed. It is expressed as the importance of having good relationships with friends, neighbours, and family members.

It's simple, like I can do everything on my own. I can do things and work in the field as much as I can at my age. I do my housework and cook by myself every day. I feel fine. I always chat with friends. We always chat and never get angry with each other. My son takes me everywhere that I want to go. He also gives me some money when I go to the temple. I feel like I'm getting blessed. My children keep visiting me. They fill my refrigerator with fish and other things. (Older person 2)

Healthy ageing means having the ability to do the activities of daily living, working and maintaining a household although needing support from others especially family members.

### Needing care from healthcare providers regardless of disease or illness

Healthy ageing means having a relationship with the healthcare providers although being free from illness or diseases. It also means living well with a disease and preventing related complications. It means being able to perform self-care behaviours such as taking medicine to prevent or treat sickness. However, at the same time, healthy ageing means to have support from healthcare providers. It means taking care of and feeling secure in life from healthcare professionals. It means having the willingness to follow a physician's guidance in dealing with any illness or disease. It means following up healthcare providers’ suggestions and feeling good when receiving their support.

When I have some pain, I am well. I wasn't upset. I knew each person has to deal with diseases but I need to see the doctor. I often go to Tambon Health Promoting Hospital to get a suggestion from nurses. If she said that's ok, it was okay. I didn't get stressed and wasn't fed up with it. (Older person 6)

Support from healthcare providers to strengthen the individual's self-care is considered important to being a healthy ager.

### Being at peace

The second category of description is being at peace, meaning having a peaceful life and a peaceful mind. It is conceived as having positive emotions, absence of stress, and being calm. It means to stop worrying. It is about knowing by own life experiences how to live and to know what life means. This descriptive category brings together two sub-categories illustrating how healthy ageing means to be at peace. These are about focusing on living in the middle way and accepting death.

### Focus on living in the middle way

Healthy ageing means being able to live in the middle way, which implies having balance in body and mind and doing things with awareness. It means balancing feelings and not enjoying individual felicity too much or worrying too much about the situations that happen in life. It means staying peaceful without blaming others as well as thinking and saying good things that cause peace.

You know, if you enjoy your happiness too much, you will lose something. You shouldn't become obsessed with enjoyment. You should be conscious and stay in the middle. It's a fact that not everything will satisfy you and you must know that. You shouldn't expect to always be pleased. You should think about things in degrees of moderation. (Older person 1)

Focus on living in the middle way indicates living a life of moderation and avoiding extremes.

### Acceptance of death

Healthy ageing means viewing death as a natural occurrence. It means accepting and being at peace with the coming death. It is about understanding that one's life expectancy is uncertain. It means confronting death because death is a natural phenomenon that everybody has to face. It implies that this is a natural event and is in line with the basic principles of Buddhism. The following quote illustrates how older persons conceptualized healthy ageing as acceptance of death:

I feel peaceful when I keep the religious rules. Nothing remains forever. When it is time for me to die, I can't stop it. I know I should accept it I'm not scared of death, though. I know I'll die when it's time for me to. I keep my heart cheerful. (Older person 11)

Healthy ageing reflects that death is a process of life that nobody can avoid. It is seen as a regular part of everyday life which means everything goes naturally. Everything changes and nothing lives forever.

### Being a valuable person

In this category older people viewed healthy ageing as being a valuable person. It means being useful to others, especially to family members, friends, society, and Buddhism. It is about contributing with good things to others. It is doing something that others find useful and are thankful for. In this conception healthy ageing was described as doing good and receiving appreciation and respect.

### Doing good

The meaning of doing good is expressed as being a good role model for others such as thinking, saying and also doing good things which will be of some advantage to others. It also means not harming others in society. The following quote illustrates how older persons conceptualized healthy ageing as doing good:

In my family, we don't yell at children. I never cheat. I, in this life condition. I believe that good health is the result of noble things. For me, I am not jealous or hate anyone. My mother was a nun and she taught me this and I do the same to my children. I tell them not to steal or commit wickedness. (Older person 12)

Doing good means being a good person and teaching this to children. It is a way of transforming good behaviour from one generation to another. Furthermore, in this subcategory, older people expressed doing good as sharing things with others such as making merit and offering food to monks. The following quote illustrates this conception:

I go to the temple every day to give food to monks. It's a forest monastery. I go to make merit. I made merit every observance day, the holy day, and I also donate flowers made from silver to the temple. Whenever other villagers arrange any religious ceremony, I join them. Whenever someone raises money, I always donate it and it's also an opportunity for me to join at the temple. When I make merit or share things with paupers, I feel good. It's the custom that I've been following. (Older person 7)

The meaning of doing good such as sharing with others included donations such as giving money for constructing buildings in the monastery, offering money, doing a charitable deed and making a donation to a disadvantaged person. It means the generosity which is a part of the doctrines of the Buddhist religion.

### Receiving appreciation and respect

Healthy ageing is seen as receiving appreciation and respect. It means to receive positive feedback from others such as children and grandchildren, as well as from friends. The confirmation from others makes the older person feel proud and happy. The following quote illustrates how older persons conceptualized healthy ageing as receiving appreciation and respect from others.

I cooked food and I gave it to my neighbours for free. I'm happy to cook for my neighbours. I'm glad when they tell me my food is tasty. I have just cooked the neighbours some mushrooms that I picked myself and they said they were delicious. (Older person 4)

This subcategory, being a valuable person can be compared with being part of a community or a group of friends. It is about thinking of others, doing something of value for others and experiencing meaningfulness and high self-esteem.

## Discussion

The findings in this study answered the questions of how older people in the Isan-Thai culture conceived healthy ageing and what the different conceptions regarding the healthy ageing of older people were in this region. The findings from this study are made up of descriptions that provide an insider's view from older persons’ experiences of their world. It is by their lived bodies that they have experienced healthy ageing and in the interviews they expressed what healthy ageing meant to them. According to Merleau-Ponty (2005/1945), the lived body is described as the body and soul being a concurrent whole. From this holistic theory, the findings of this study can be referred to and demonstrated as a holistic understanding of healthy ageing as the physical, mental, social, and spiritual aspects of healthy ageing have all been considered as parts of a single entity.

The first category of the findings shows that the informants conceptualized healthy ageing as being independent in dependence. This finding consisted of being able although requiring support and needing care from healthcare providers regardless of diseases or illness. It can thus be seen that the meaning of healthy ageing is related to others. Healthy ageing is to live an independent life, although dependent on family members, friends, and others. Moreover, healthy ageing involves needing support and feeling secure which is related to healthcare providers. The scope of healthy ageing in the Isan-Thai culture shows similarities with western countries concerning the issue of independence (Hansen-Kyle, 2005). Similarly, Liu, Beaver, and Speed (2014) remarked that being healthy related to one's ability to perform self-care behaviours and ability to manage an individual's life in order to maintain health. However, the understanding of healthy ageing among older people in the Isan culture was different from those in other studies concerning being dependent. This finding is similar to the previous studies by Danyuthasilpe et al. (2009), who studied healthy ageing in the northern region of Thailand. Healthy ageing was described in terms of being able to do, or choose something meaningful and having a harmonious family. One can clearly see that the importance of independence is highly valued in western countries (Hansen-Kyle, 2005; Ingersoll-Dayton, Saengtienchai, Kespichayawattana, & Aungsuroch, 2001; Roe, Whattam, Young, & Dimond, 2001; Thanakwang, Soonthorndhada, & Mongkolprasoet, 2012). However, it may not be as important in other cultures because independence is a cultural value (Hansen-Kyle, 2005). In Thai culture, especially in the Isan cultural context, older persons have historically received care from their family members (Knodel & Chayovan, 2008; Wongsawang, Lagampan, Lapvongwattana, & Bowers, 2013). Children play the role of repaying their parents for taking care of them during their own childhood dependence and provide economic support, take care of and live with their parents. It is a key attribute of Buddhist philosophy and it is related to interdependence (Choowattanapakorn, 1999; Danyuthasilpe et al., 2009). This mutual relationship can be related to Merleau-Ponty's (2005/194) theory of intersubjectivity and as the reciprocal nature of perceiving another like oneself.

The findings in this study reflect the care and support from children, friends, and healthcare providers that are very important for older persons. However, modernization effects the lifestyle of people in Isan as family members in Isan live separately. Family sizes are getting smaller because family structures are changing (Knodel & Chayovan, 2008). The care of older persons by their children is most likely to change in the future. Furthermore, older people may risk isolation due to loss of a spouse and close friend (Ness, Hellzen, & Enmarker, 2014). The older population who live alone may increase. Therefore, public health policy should pay attention to providing a foundation for long-term care among older people who do not have the relatives in order to promote healthy ageing. Healthcare providers should concern a group of oldest old population (over 80 years) because this group is expected to effect on changing in their ability to perform activities to take care of themselves (Lundin, Berg, & Muhli, 2013). For this reason, the oldest old may require more services and need more support of healthcare providers when compared to other groups of older people. In order to promote healthy ageing for the whole older population in Isan, healthcare providers should pay attention not only to older people who live with chronic diseases but also to support those who are free from chronic diseases to perform self-care and promote healthy ageing.

The second category, “being at peace,” consisted of having focus on living in the middle way and acceptance of death. Participants in this study suggested that healthy ageing is to stay peaceful and to avoid the extremes of life. It means living a calm life, having good emotions, and the absence of worry. Furthermore, it means accepting the coming death and being able to confront death. This conception of healthy ageing in Isan culture corresponds well with the findings of Danyuthasilpe et al. (2009), who described the meaning of healthy ageing as having a peaceful mind. The findings in this present study are also related to Buddhism where the principle at the very heart of Buddhism is tranquillity and peace (Yeh, 2006). Buddhists achieve tranquillity and peace by learning to see through the perception of what reality is through the true nature of things, and thus true reality exists (Thathong, 2012). Moreover, this category can be linked with the concept of spirituality and health (Chiu, Emblen, Van Hofwegen, Sawatzky, & Meyerhoff, 2004). Spirituality, which is focused on mediating peace and calmness, is related to emotional well-being (Pincharoen & Congdon, 2003; Rattanamongkolgul, Sritanyarat, & Manderson, 2012).

In the third category, healthy ageing means being a valuable person. Healthy ageing is related to doing good for others such as being a good role model, doing charitable deeds with joy and making merit. Furthermore, the participants indicated that healthy ageing is to receive appreciation and respect. This way of understanding healthy ageing is related to the study by Thanakwang et al. (2012), who showed that healthiness is deeply connected with religious belief. When they do something good, older people feel satisfied. One can easily see how this category is connected to Buddhism, where, Karma is the notion of action in which good actions are called merits (Yeh, 2006). Doing good deeds, or making merit, is a moral imperative in Buddhism. Merit making is a key concept within Buddhist thought. In Thailand, it can be seen that religion plays an important role for older people. Older people practice Buddhism. They believe that merit will mean a better life in the future. In this case, a better life may mean having power, being rich or in good health (Choowattanapakorn, 1999). This also corresponds well with the healthy ageing model (Thiamwong et al., 2013) which was carried out in southern Thailand. That model comprised three themes: normality, nature, and dharma (Thiamwong et al., 2013), and are connected to the teachings of the Buddha. The older people in the present study also stated that healthy ageing means receiving appreciation and respect. This finding can be linked with the Thai tradition and Buddhism where older persons are highly respected and honoured by people in their family and the society (Browell, 2000). Another possible explanation may be related to older people's dignity of merit (Nordenfelt, 2003). This can be described as how older people feel about themselves and is related to an individual inner self. Older people have done a lot of things in their life. They have had significant achievement in their life. They have their own experiences. For these reasons, they are respected and have received appreciation which is related to their self-esteem. The importance of self-esteem is associated with physical health and psychological well-being (Neff, 2011) and seems to contribute to healthy ageing.

### Methodological considerations

Trustworthiness within phenomenographic study refers to the truth value of the findings and a description of phenomenon which corresponds to participants’ experiences of the phenomenon (Åkerlind, 2012). In this study, the research process is described in each part of the study to make the research procedure transparent. The findings came out in a limited number of categories, called categories of descriptions. A set of the categories created the outcome space which is related to the data (Marton & Booth, 1997). The authors have attempted to describe the relationship between the data and the categories for describing ways of experiencing healthy ageing. The categories of descriptions are relevant to illustrative excerpts from the transcripts. The categories of descriptions were confirmed through discussion with the authors’ team to reduce bias therefore improving credibility (Polit & Beck, 2012). Another aspect of a present study's trustworthiness is transferability (Polit & Beck, 2012).

The findings may be transferred to other older people regarding the conception of healthy ageing in the Isan region. However, it is necessary to consider that this study includes a group of older people from only one province in the Isan region of Thailand. The transferability of this study is therefore limited. In this study 17 older people participated, the number of participants considered to be sufficient for variation conception of healthy ageing to emerge. However, the variation of participants was limited. Fewer men than women were interviewed, so the issue of healthy ageing for men may not be fully represented. Older people who had a psychiatric diagnosis were excluded and consequently, the conceptions of healthy ageing for this group were lost. Furthermore, all of the participants believe in Buddhism, which is grounded in their belief and understanding and may influence the findings regarding the conception of healthy ageing. Although, the main religion of Thailand is Buddhism, there are diverse religious beliefs in the country and the conceptions of healthy ageing might differ in those groups of different religions.

## Conclusion and further research

In this study, healthy ageing in the Isan-Thai culture is a phenomenon that is related to the individual, family, society, healthcare providers, and religion. Like others, the concepts of healthy ageing in this study were being at peace and being a valuable person which are influenced by religion. However, in this study healthy ageing was also conceived as being independent in dependence. This study highlights the fact that healthy ageing should be viewed from a culturally sensitive perspective if one is interested in fully understanding the concept. Healthcare providers should consider cultural variations when healthy ageing is conceptualized in order to implement a holistic view to promote health among the senior citizen. Future studies should be designed to integrate different groups of older people from various settings. Moreover, based on the findings in this study, the conception of healthy ageing should also be studied from the perspective of relatives and healthcare professionals as they are responsible for the care of older people in the Isan region.
